# Eye Injuries, with Special Reference to the Workmen's Compensation Act, 1906

**Published:** 1909-11-13

**Authors:** A. Maitland Ramsay

**Affiliations:** Ophthalmic Surgeon, Glasgow Royal Infirmary


### Hospital Clinics.

### EYE INJURIES, WITH SPECIAL REFERENCE TO THE WORKMEN'S COMPENSATION ACT, 1906.

By A. MAITLAND RAMSAY, M.D., Ophthalmic Surgeon, Glasgow Royal Infirmary.

(The Opening Address of the Paisley Medical Society, on November 11, 1909.)

The beginning of what may be termed modern State regulation of the relations between employer and employed dates from the beginning of the nineteeth century, when an Act was passed imposing on employers certain obligations for the better housing, clothing, and education of apprentices. From this small beginning legislation has, in more recent years, advanced with rapid strides, and the duties and responsibilities of employers have not only been more explicitly defined, but have been greatly extended. The Employers' Liability Act of 1880 went merely so far as to extend to the workman the general principle that personal fault or negligence on the part of the master rendered him liable in damages for any injury sustained by an employee; but if, in consequence of carelessness on the part of a servant, an accident occurred to a third party, no liability attached to the master. It was, therefore, quite a new departure when, under the provisions of the Workmen's Compensation Act of 1897, the employer was made liable for compensation for personal injuries by accident to an employee, though no one could be held to be at fault. The benefits of this Act were at first restricted to certain specified trades, but in 1900 they were extended so as to include agricultural labourers, and the Workmen's Compensation Act of 1906, which repealed the other two and took their place, is so wide in its scope that it practically affects, or may affect in some way or other, every class of the community.

For the successful working of the Act the co-operation of medical men is indispensable; and it is therefore important that every doctor should make himself familiar with its chief clauses. Briefly put, the main provision is that if an employee receives an injury "arising out of and in the course of the employment" the employer is legally bound to pay him half his weekly wages and to continue this payment until he is able to resume work. The Act does not provide any solatium for personal loss, nor does it, as Sheriff Glegg has recently pointed out, guarantee or ensure re-employment after injury. Unless the workman be disabled for a week he cannot make any claim; but if the results of his injury incapacitate him for more than that time, he can claim compensation, commencing from the day on which he was hurt. The Act also covers disablement arising from certain scheduled diseases, termed industrial diseases, among which may be mentioned miner's nystagmus and bottlemaker's cataract. If a workman contract one of these in the course of his employment he can claim compensation in exactly the same way as if his incapacity had been caused by an accident. All employers are liable under the Act, and all workers (sailors, agricultural labourers, and domestic servants included) are entitled to compensation, provided that the total sum of their weekly earnings does not amount to more than £250 in the course of a year. The Army, Navy, and the Police Services are excluded from the benefits of the Act, as are also outworkers, *i.e.* persons to whom articles or materials are given out to be made up, cleaned, washed, etc., in their own houses, or in other premises not under the control of the person who gives out the material.

The design of the framers of the Act was, undoubtedly, to minimise the need for legal procedure; but their purpose in this respect has not been achieved, for no statute of recent times has been more provocative of litigation. In the Sheriffdom of Lanarkshire, during 1908, there were 678 arbitrations before the sheriffs and 2,678 memoranda recorded. A considerable proportion of these were connected with cases arising from injuries to the eye, accidents of this nature being frequent causes of dispute, because, on account of the great delicacy of the ocular tissues, they are so often followed by deterioration or loss of vision. If a wound heals perfectly, the presence of a scar in any other part of the body is not, as a rule, of much consequence; but in the eye, if the functional integrity of the organ is to be perfectly preserved, there must be no scar at all.

In the cases that come before the Courts the issue is frequently decided on the medical report, and as the surgeon who has examined a case may be cited to a Court of Law to give expert evidence, it is of the greatest importance that he carry out his examination from the very first in a methodical and systematic manner. He ought also to take full notes at the time, and preserve them carefully for future reference, because it is the original notes, and not a copy, that he will be allowed to use to refresh his memory in the witness-box. The patient ought to be examined as soon as possible after the accident, before he has had time to recount his symptoms to companions, who may tempt him to exaggerate, or even to deceive. It is always well in the first place to ascertain clearly all the facts of the case—exactly how the accident happened, what caused the injury, and, if the cause be a body projected from without, then the nature of the missile, the direction and distance from which it could have come, and the probable velocity with which it was travelling. The tension of the eyeball ought next to be estimated and the vision tested. Afterwards there should be a careful examination, in regular order, of the orbital margins, the eyelids, the conjunctiva, and the cornea; and diligent search should also be made for any sign of perforation of the globe. The colour and general appearance of the iris, and the position, size, shape, and mobility of the pupil should be noted and contrasted with those of the sound eye. Unless the presence of blood in the anterior or posterior chamber prevent it, an ophthalmoscopic examination should never be omitted, for by this means any alterations in the lens and vitreous, and in the deeper structures of the eyeball, can at once be detected. It will be of the greatest advantage to the surgeon to have some knowledge of the trade of the workman on whose injuries he is requested to report. It is all important for him to be sure of his facts, and to be in a position to make his examination as exhaustive as possible; for the penetrating art of cross-examination may find out omissions and discrepancies where none were supposed to exist. It is usually the aim of the opposing counsel to show up imperfections and contradictions in the medical testimony, and the conflicting nature of expert evidence is often alluded to by lawyers. An eminent sheriff, now deceased, once told me that he frequently added to his interlocutor that the " medical evidence in the case was, as usual, very unsatisfactory." In this connection, however, it must be remembered that a medical opinion cannot be exact in the sense in which a legal one is, for in law there is no personal equation. In medical matters, on the other hand, everyone will admit that one person may be much more seriously affected by any given injury than another. There can never be absolute certainty. An expert opinion can only be founded on the doctor's knowledge of the facts, and his personal experience of similar cases, and whenever several causes may contribute to bring about a result, a dogmatic statement on oath is impossible. The doctor can only say what in his opinion is likely to be the case; he cannot be certain. Moreover, the difference in the statements in medical reports is often more apparent to the layman than it is to a doctor, whose professional knowledge may enable him to reconcile statements which at the first glance seem contradictory. The unsatisfactory character of medical evidence would therefore be less frequently commented on if medical men were careful to make sure of their facts and to write their reports in clear, simple language, avoiding as far as possible all technical terms. If they would merely, for example, describe a man as having a black eye, and not write of him as suffering from subconjunctival ecchymosis or of extravasation of blood beneath the cuticle. The object of a medical witness is not to win a case for the one side or for the other, but to state the truth, the whole truth, and nothing but the truth, in order that by his testimony strict justice may be meted out to master and servant alike.

The liability of the employer is so well defined by the Act that, after an accident has been duly reported, the recognised amount of compensation is usually paid at once, and continued regularly till the workman is able to resume his employment. It is only after he is thought to have recovered from the effects of the injury and refuses to go back to work, or when, after six months' incapacity, a dispute arises over the amount of the lump sum the employer is entitled by the Act to offer in lieu of a continuance of the weekly payments, that the services of the Court or of an arbiter are necessary. The medical expert may then be called upon to say whether the injured man is fit for work, or, if not, to give an opinion as to whether the results of the injury are likely to be temporary or to lead to permanent incapacity. He may also be asked to say what kind of occupation he thinks the workman can follow, or to determine the loss to his earning ability, which has resulted from the accident. In this connection, however, it must be remembered that a man's capacity for work is not synonomous with his ability to earn wages. A man may be physically quite fit for work, but he may be unable to find employment. His earning ability, therefore, depends not only upon his health, but also upon his technical skill, as well as upon the state of the labour market. The assessment of compensation for partial incapacity is really a matter for the Sheriff; but, naturally, he expects guidance from the medical expert regarding not only the kind of employment the workman can undertake, and the conditions under which he can work with safety, but also the degree to which his technical skill has been lessened in consequence of the injury, and what probability there is that he will in time recover his former capacity. The surgeon will also be expected to say how far the results of an injury have been modified by pre-existing disease. He must distinguish clearly between lesions that have previously existed in an injured eye, and those which are directly the result of an accident.

Sym has proposed that every skilled workman should provide himself with a " health ticket," on which, in addition to other details, the actual state of his vision would be recorded, and, as a matter of fact, since the Act was extended to seamen, every sailor who ships from Glasgow is medically examined before he is engaged. If, however, such a strict examination were to be insisted upon in all cases, and especially by insurance companies, many a man suffering from a physical defect which does not necessarily incapacitate him from work, would soon find himself in the ranks of the unemployed. Indeed, such an arrangement would only be practicable if the Act were amended so as to permit much greater latitude in contracting out, and if closer relations were entered into between the State and the great Friendly Societies. At present the financial resources of the latter are being very seriously drained by the great increase in the number of claims since the passing of the Act of 1906. Most masters might be trusted to look well after the interests of their own aged or infirm servants; but, as the the law now stands, they are naturally unwilling to employ anyone who is maimed, or who is not physically fit for work. It is undoubtedly the case that many employers are showing a decided disinclination to give work to elderly men, because they look on them as, from their age. less active, and so more liable to accident. The financial responsibility is too great, and insurance companies (which must obviously be conducted on strictly commercial lines) are quite alive to the fact that it does not pay to insure a potential cripple. Consequently any workman who does not come up to their requirements, whether through age or otherwise, is put on a prohibited list which is distributed among the employers, and any master who engages a workman whose name appears on that list does so at his own risk. This is a serious matter in more ways than one. First, the absence of older men from shops and works involves loss of guiding skill and experience, and undoubtedly lessens the steadiness and moral strength of tone, which are primary essentials in such establishments, and make so much for good discipline; and, second, as regards the general difficulty that the physically less fit have in finding employment under the new conditions, it is stated on excellent authority that increasing numbers of these workers are being compelled to apply for such aid as the Poor Law can give for their wives and dependents.

A man may have an eye the sight of which is congenitally defective, and yet know nothing about it until dimness of vision makes its appearance after some injury which in itself may have been very trivial. In these circumstances careful examination may reveal the existence of a high degree of astigmatism or of myopia, or of pre-existing disease of the optic nerve, and so all doubt as to the real cause of the defective sight will be completely removed. Then again, an injury in itself trivial may become very serious when complicated by blennorrhœa of the tear passages, and the healing process will always be retarded by such general diseases as glycosuria or albuminuria, either of which may of itself give rise to grave disturbance of sight. If, then, a workman, suffering from either of these disorders, happens to meet with an injury which is followed by defective vision, he will naturally attribute the failing sight to the injury, and claim compensation for a condition arising from the disease and not from the accident.

The following are a few personal experiences of the difficulties that may present themselves to the medical expert in writing a report, or in giving evidence in a case where the effects of an injury have been modified by acquired or by constitutional disease. I have known a chancre of the lid to be caused by the well-intentioned efforts of a workman suffering from syphilis to remove with his tongue a foreign body from the eye of a companion. I have seen a man lose an eye through gonorrhœal infection following on a comparatively trivial injury, and in patients affected by hereditary syphilis it is by no means uncommon to find severe interstitial keratitis supervene on some slight wound. In high myopia the results of accident are often much more serious than they would be in a normal eye. I remember a man, on whose case I was asked to report, who said that when stooping to get something out of a box the right side of his forehead accidentally came in contact with a projecting shelf. The injury in itself was exceedingly slight, but the patient was highly myopic, and the result was a detachment of the retina, which gradually increased and gave rise to permanent blindness of the right eye. Malignant disease is frequently attributed to injury, and a sarcoma of the choroid is often supposed by the patient to be of traumatic origin. The medical expert, however, in such a case would have to point out that the condition could not arise from the injury alone, which at most could only be an exciting cause in a person predisposed to the disease. Lastly, I recently saw a man in whose case an injury to the forehead had been followed within a day or two by complete ptosis, and later by paralysis of all the branches of the oculo-motor nerve on the same side. The patient naturally attributed the symptoms to the injury, but careful examination revealed the presence of the Argyll-Robertson pupils, atrophy of the optic nerves, and absence of the patellar reflex on his right side. In this case the injury at most accelerated the onset of the oculo-motor paralysis, which was really merely a symptom of pre-existing tabes dorsalis.

In recent years, various attempts have been made by different authorities to express by means of a mathematical formula the loss to the earning ability occasioned by visual defects; but as Evans justly observes, " no formula, however complicated, could possibly include such important factors in the estimation of compensation as the intelligence and perseverance of the patient, the work, the state of the labour market, the prejudices of employers and employed, etc.," and yet, if a just decision is to be arrived at, these are factors which cannot be overlooked.

In the estimation of the damage to sight from the economic point of view, the most important factors to be considered are the acuity of vision, the field of vision, binocular vision, and muscular defects.

The visual acuity is the most important. This is measured by the patient's ability to read standard test types at a given distance. The types in general use are those devised by Snellen, who constructed them on the basis that for the normal eye the minimum visual angle amounts to about one minute. The test consists of letters of varying size, arranged in rows, the strokes of which the letters are formed being in every case one-fifth of the diameter of the letter, which at the regulation distance is therefore seen with a visual angle of five minutes. Above each row is a number showing the distance in metres or feet at which the row should be recognised. The types must be placed in a good light, and in practice it is most convenient to place the patient at a constant distance from them. This distance is fixed at 6 metres or 20 feet, because the rays coming from an object are then, for all practical purposes, parallel, and the examination is thus not complicated by the accommodation. It is often more convenient, however, in testing those with very defective sight, to employ a simpler test object; for example, to ask the patient to count the outstretched fingers held against a black coat. If he cannot do so, the surgeon should try if he can distinguish hand movements, and if he again fail, trial should be made if a light can be distinguished when held in front of the patient's eyes. In carrying out the test with Snellen's types, any error of refraction must first of all be corrected, since an ametropic eye without glasses gives the relative and not the absolute acuity of vision, and consequently the result is no measure of its general usefulness. The examination of the visual acuity is completed by asking the patient to read small type, and, if necessary, spectacles suitable for his age and refraction must be used.

It is a great convenience to express visual acuteness in a fractional form ($V = \frac{d}{D}$), for this method is employed and understood by ophthalmologists all over the world. When recording visual acuity in a medical report, however, it is very misleading to look on this form as an ordinary vulgar fraction, and to say, for example, that $\frac{6}{12}$ or $\frac{6}{24}$ is equal to a half or a fourth of the normal. A layman, reading such a statement as that, would inevitably come to the conclusion that the patient's vision for his work is much worse that is actually the case, for it should be remembered that what is regarded scientifically as normal vision is determined according to a higher standard than is required for efficient working or wage-earning acuity. The farm labourer can perform his duties quite satisfactorily although he has much less sight than would be necessary for a skilled mechanic. It would simplify matters if a list of employments could be drawn up, with a corresponding list of the minimum visual acuity required for each, similar to the visual requirements published for the Army and Navy; but obviously this is not practicable. We must content ourselves, therefore, by dividing all workmen into two main classes—first, those whose avocation demands a high degree of visual acuity—for example, professional men, skilled mechanics, etc.; and, secondly, those who from the nature of their employment can work satisfactorily with a less degree of visual acuity—for example, carters, labourers, etc. Even for the first of these, however, the visual standard required is lower than that acknowledged to be the normal scientific standard. Würdemann has suggested that the visual demands of the first class should be fixed at from $\frac{3}{4}$ to $\frac{1}{7}$ of the scientific standard, and of the second class at from $\frac{1}{2}$ to $\frac{1}{20}$; but even if one accepts this wide range as a working basis, individual exceptions will constantly be found. The minimum acuity must always vary within wide limits. Fergus has cited a number of cases where men with a very low degree of visual acuity have earned full, or nearly full, wages as colliers, iron-turners, boiler-makers, blacksmiths, engine-fitters, etc.

I remember a miner who was so blind from choroido-retinitis that he had to be led by his son to his work; yet he earned full wages. This, however, was before the Act of 1906, and, undoubtedly, the man exposed himself to risks which were unjustifiable. Every practitioner must know of cases where the eyes have been damaged in early childhood, and yet the persons concerned, with a very inferior visual acuity, are doing work which, according even to Würdemann's classification, should be beyond their power. After all is said, however, it must not be forgotten that, in the case of a young person determined to follow a certain avocation, the patient's age, his brain power, his character, and his perseverance are factors which are specially important; but in the case of a man who, accustomed to work with good sight, suddenly suffers marked deterioration of vision, as the result of an accident, the conditions are quite different, and the same factors do not have the same value.

The field of vision can be mapped out accurately only by the help of a perimeter; but the surgeon can obtain much information by the simple means of measuring the field roughly with his hand and comparing that of the patient with his own. When, however, a case is likely to be taken into court, the medical expert should make a full chart, which can be produced if necessary and demonstrated to the Sheriff. The entire field of vision is divisible into a central and a peripheral portion. The former is represented by a very small part of the whole, and its size corresponds roughly to that of the thumb nail held at arm's length. If, however, this small area be destroyed, the patient is quite unable to read, although he may be able to see his way about with comparative ease. An injury, therefore, which would ruin a clerk or an engraver might leave the unskilled labourer little damaged as far as his working capacity is concerned.

Dehenne and Bailliart have pointed out that peripheral restriction of the visual field is relatively of more importance to coachmen, labourers, etc., than it is to those who, like skilled mechanics, require a higher standard of visual acuity for their work. This fact should be borne in mind when one is trying to estimate the loss of earning capacity. Another fact of equal importance, in the same connection, is that two-thirds of the binocular field belong to both eyes, so that even if one eye were blind, the restriction in vision would amount to only about one-sixth of the whole. Loss of the temporal or nasal portions of each field does not interfere much with working capacity, but homonymous hemionopsia impairs the earning ability according to the patient's occupation and according to whether or not it is accompanied by diminution of the visual acuity or by loss of binocular vision.

Binocular Vision.—The value of the two eyes together is very much greater than the sum of the value of each separately. Dehenne and Bailliart have classified all workmen according to whether they require good binocular vision or an extensive field of vision, and place in the first category all those who, when they become presbyopic, require glasses for their work. On binocular vision depends, to a large extent, our ideas of solidity and distance, and in many occupations it is, therefore, of the first importance. The simple test devised by Roche, or the diaphragm test recently described by Bishop Harman, afford a very simple method of demonstrating the presence or absence of binocular vision; but there are other means, such as Snellen's test or the ordinary stereoscope, which may be employed. Binocular vision must clearly be at once lost when a patient becomes blind of an eye; and its economic value has been much discussed in connection with those cases where a workman has lost an eye through accident. The Workmen's Compensation Act does not, as has been already said, indemnify a worker for the loss of his eye, or, indeed, for any physical damage he may sustain, but merely makes adequate pecuniary provision for him during the time he is unfit for his employment. The question, therefore, which is frequently debated in the Law Courts is whether a man with one eye is as fit for work as a man with binocular vision. The just answer to that question depends both upon the man himself and upon the nature of his work, and, consequently, each case must be considered on its own merits. In every trade numerous examples of one-eyed men earning full wages are to be found; but all of them will admit that, to begin with, they experienced more or less difficulty in estimating the position of objects, and in calculating distances. To some workmen the adapting of themselves to the altered circumstances seems almost like a new apprenticeship; some, owing to their temperament and perseverance, overcome the difficulties in a very short time, while others require considerably longer, and a few, owing to nervousness and faults of character, never attain proficiency under the new conditions. The difficulties must obviously always remain greater if a man has to work among machinery, or on scaffolding, or underground, or where the light is insufficient. From the employer's point of view the man with one eye does undoubtedly run more risk of injury than the one with two, and consequently, in estimating the wage-earning ability of the one-eyed man, this prejudice on the part of the master ought always to be taken into account. Provided the workman possesses two good eyes, the accidental loss of one in no way impairs the visual acuity, and only slightly restricts the visual field. The diminution of working capacity, therefore, depends almost wholly on the loss of binocular vision.

Würdemann says that one year is a sufficiently liberal allowance of time to give to anyone for the adaptation of monocular vision to the demands of his work, and states that "approximately a one-eyed person has lost thirty per cent. of his earning ability for the first year after the accident, and twenty per cent. afterwards for the higher class of trades, and for the lower class the proportion would be twenty-seven per cent. for the first year and eighteen per cent. thereafter." If blindness of one eye develop gradually the workman has time to adapt himself to the changes incident to the loss of binocular vision, and if he has lost an eye in early childhood he grows up to be practically unconscious of his defect; but it is a very different matter when the loss of sight on one side occurs suddenly in adult, or more especially in advanced life, and naturally these are the cases which most frequently give rise to claims for compensation.

A question occasionally arises as to the relative value of one eye over the other. Statistics prove that the left eye is the one more frequently injured, and workmen themselves attach more importance to the right eye, probably, as Evans has suggested, because most of them are right-handed. A carter, however, would form an exception, because when his cart is loaded he always leads his horse on the near side, and naturally turns to the left when he hears vehicles approaching from behind, hence the loss of the left eye is a more serious matter for him than the loss of the right would be.

Paresis or paralysis of an ocular muscle causes double-vision, and occasionally arises from accident. As long as the diplopia persists the patient is unfit for work; and if he tries to adjust matters by covering up one eye he puts himself in exactly the same position as if he were a one-eyed man.

*(To be continued.)*

